# A Low-Cost Non-Intrusive Method for In-Field Motor Speed Measurement Based on a Smartphone

**DOI:** 10.3390/s21134317

**Published:** 2021-06-24

**Authors:** Paula Paramo-Balsa, Juan Manuel Roldan-Fernandez, Manuel Burgos-Payan, Jesus Manuel Riquelme-Santos

**Affiliations:** Department of Electrical Engineering, Universidad de Sevilla, 41092 Sevilla, Spain; pparamo@us.es (P.P.-B.); jmroldan@us.es (J.M.R.-F.); jsantos@us.es (J.M.R.-S.)

**Keywords:** induction motors, audible noise, rotational speed measurement, non-invasive speed measurement, contactless speed measurement, in-field speed measurement, in-field load estimation

## Abstract

Induction motors are broadly used as drivers of a large variety of industrial equipment. A proper measurement of the motor rotation speed is essential to monitor the performance of most industrial drives. As an example, the measurement of rotor speed is a simple and broadly used industrial method to estimate the motor’s efficiency or mechanical load. In this work, a new low-cost non-intrusive method for in-field motor speed measurement, based on the spectral analysis of the motor audible noise, is proposed. The motor noise is acquired using a smartphone and processed by a MATLAB-based routine, which determines the rotation speed by identifying the rotor shaft mechanical frequency from the harmonic spectrum of the noise signal. This work intends to test the hypothesis that the emitted motor noise, like mechanical vibrations, contains a frequency component due to the rotation speed which, to the authors’ knowledge, has thus far been disregarded for the purpose of speed measurement. The experimental results of a variety of tests, from no load to full load, including the use of a frequency converter, found that relative errors on the speed estimation were always lower than 0.151%. These findings proved the versatility, robustness, and accuracy of the proposed method.

## 1. Introduction

Electric motors are extensively used as drivers of a large variety of equipment in industry, services and even household appliances. As a result, nearly half of the electric energy produced worldwide is converted into mechanical energy by means of electric motors and integrated as added value into the process or final application. Almost 90% of industrial drives are based on three-phase squirrel-cage induction motors [1,2]. Therefore, the ways in which induction motors are selected, used, controlled, and managed have a great influence both on the electric energy bill for consumers (industrial or domestic) as well as on energy consumption, the volume of CO_2eq_, and other polluting emissions for society.

A proper measurement of motor rotation speed is a key component of most industrial drives in order, for example, to control the process, monitor the performance or identify the main parameters of the motor (or drive). As an example, the measurement of rotor speed is a simple and broadly used method in industry to estimate the motor’s efficiency or its mechanical load [3,4]. For continuous speed monitoring, the coupling of a tachometer to the motor shaft is often used. On other occasions, an alternative (more sophisticated and expensive) method of rotor speed estimation, by means of on-line analysis of the input current, is also used [5,6,7]. For occasional speed measurements a portable optical tachometer can be used, although this solution often requires sticking a reflective adhesive strip on the motor shaft. This kind of situation takes place typically during audit (monitoring) periods of the plant, when the performance of induction motors must be evaluated a few times a day (hourly) or over a few days. In such cases, the estimation of the mechanical load and/or the efficiency of the motor, based on rotor speed measurement, is a common solution since the method combines acceptable precision with low cost. For those cases, the use of a portable optical tachometer is often the preferred choice. Unfortunately, sticking the reflective strip on the motor shaft is not always possible since the shaft is not always accessible (for safety reasons, or because the shaft coupling is protected by a cover) or visible (motor inside a housing). 

There is a broad collection of published papers on rotational (and linear) speed measurement techniques, including contact and contactless sensors, relying on sensors based on optical reflection, and electromagnetic field fluctuations and conductive strips. A contact-type synchro was used in [8] as a primary transducer where a rotating magnetic field was used to measure rotational speed with high accuracy. The principal applications of contactless electrostatic sensors and correlation signal processing techniques to real-time measurement of rotational speed are presented in [9]. The results of the work suggested ‘that the distance between the electrodes and the surface of the rotating object is a key factor affecting the performance of the measurement system’. Another electrostatic sensor to measure the speed of rotational equipment under a condition of high temperature and heavy dust was also proposed in [10]. Nevertheless, although the electrostatic sensor is contactless, the measurement method remains rather invasive.

Although vision-based measurement systems were originally developed for special applications, their use is growing rapidly because of the increasing affordability and capability of cameras. An overview of vision-based measurement from the perspective of instrumentation and measurement (metrological) was developed in [11]. A rotational speed measurement system based on a low-cost imaging system was proposed in [12]. The method requires sticking a simple marker on the rotor shaft and using a commercial webcam to capture the rotation of the shaft at a maximum rate of 30 frames per second. Another nonintrusive webcam-based tachometer was also proposed in [13] as a vision-based rotor speed measurement system. The authors stated that the proposed tachometer can be used, for example, in industrial electrical energy audits to continuously estimate the load variation in three-phase squirrel-cage induction motors using the slip-based method. Vision-based measurement methods are contactless; however, the motor shaft must be in the field of observation of the camera in order to capture its movement. 

The interaction between alternating currents and electromagnetic fields that takes place in an induction motor leads to the production of the desired electromagnetic torque. Nevertheless, and as a side effect, that same interaction between alternating currents and electromagnetic fields also gives rise to the production of unwanted mechanical forces in the air gap between the stator and the rotor. These unwanted radial and axial forces lead to mechanical vibrations which are spread and transmitted through the whole motor frame and the adjoining mechanical structure. The amplitude of motor vibration at any point of the motor depends on the combination of the magnitude of the mechanical forces and the mechanical response of the motor frame and the adjoining mechanical structure [14]. Techniques for motor condition monitoring have widely taken advantage of the analysis of motor vibrations [15]. However, the vibration of motors has also been used to measure rotor speed [6,16], since the mechanical vibration also contains a component related to the rotation speed.

As mentioned previously, the interaction between alternating currents and electromagnetic fields leads to the production of the desired motor torque and additional mechanical forces in the air gap which drives mechanical vibrations. However, in addition, mechanical vibrations finally excite the surrounding air, resulting in the emission of audible motor noise. These acoustic signals have been recently used by Glowacz et al. for fault diagnosis applied to angle grinders and electric impact drills [17]. However, the motor noise, like the mechanical vibrations, must also contain a frequency component due to the rotational speed which, to the authors’ knowledge, has thus far been overlooked for the purpose of speed measurement. 

Nowadays, mobile computing technology is suffering a rapid development thanks to multi-core chips and larger memories. Thus, in a wide variety of applications, smartphones are used instead of other types of sensors since they provide several advantages [18], including the low cost of equipment, that they are widely connected to the Internet, and that their number is continuously increasing. Smartphones have different sensors such as camera, microphone, accelerometer, or GPS. This opens an opportunity to use these devices to develop sensor systems capable of monitoring application areas as different as agriculture [19], human health [20], or road traffic [21], among others. In this work, a new, low-cost, non-intrusive method for in-field motor speed measurement, based on the spectral analysis of the audible motor noise is proposed. The motor noise is initially acquired using a smartphone or a web microphone, for example, and sent to a PC by means of a wired or wireless link. A MATLAB-based algorithm, especially developed for this application, loads the data file and determines the harmonic spectrum of the noise signal, from which the motor’s rotation speed is finally estimated. The new method can be used for measuring (and eventually recording) the motor’s rotor speed over time. It is very simple and easy to use since the motor noise is broadly available and does not rely on motor parameters. The proposed method is contactless, completely non-intrusive and can be used for in-field applications without any interruption or alteration to the motor’s service.

The remainder of the paper is organized as follows. The theoretical background of the proposed method for measuring the rotor speed of a motor by means of the spectral analysis of the audible noise is introduced in Section 2. The main results of the tests performed to experimentally validate the method are presented in Section 3, where the advantages of the proposed method will also be shown. A brief discussion of the method is presented in Section 4. Finally, Section 5 summarizes the main findings of the work. 

## 2. Materials and Methods

**Figure 1:**
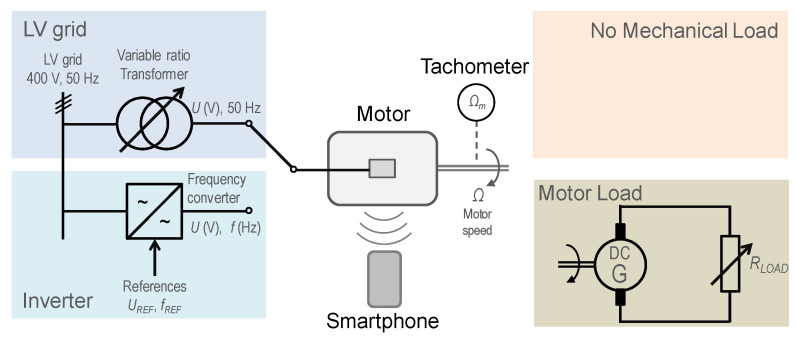
Sketch of the configuration of the different tests carried out on the induction motor.

The proposed method is based on the use of a smartphone to acquire and record the audible noise emitted by an induction motor, by means of two free apps downloaded from a web store. An educational electric machines test was used. Figure 1 outlines the test bench and the configuration of the different tests carried out on the induction motor. As can be seen, the core focus of the test bench is the induction motor, whose noise is acquired and recorded by means of a smartphone. That induction motor can be fed either from the network through a variable ratio transformer, which allows for the adjusting of the supply voltage, or by means of an inverter, which allows for the adjusting of both the voltage and frequency supplied to the motor. On the mechanical port, the motor shaft can be coupled either to a separately excited direct current generator, which feeds a variable resistive load allowing the adjustment of the motor load torque, or it can be left free, without mechanical load. A portable optical tachometer was used to measure the rotation speed of the motor shaft by a conventional and well-established contactless method, while a low-performance smartphone was used to acquire and record the motor noise. In order to improve the determination of the actual speed of the motor, *Ω_m_*, along all the tests, the motor speed was measured twice by two different tachometers and the average value of the two speed measurements was considered as the actual rotor speed, *Ω_m_*. Appendix A includes details on the audio recording apps, smartphones, tachometers, and test bench used in this work.

Once the appropriate test configuration was decided upon, that is, the voltage source of the motor (variable ratio transformer or inverter) and the mechanical load (DC generator with a load resistance, or no mechanical load at all), the tests on the laboratory bench were performed. After setting the proper voltage source and mechanical load, the voltage (and frequency in the case of inverter) and the load resistance (in the case of DC generator) were adjusted to reach the desired conditions of load and shaft speed. Once the value of the rotor speed was verified, a smartphone placed just in front of the nameplate of the motor was used to acquire and record the motor noise by means of the apps. The recorded *.wav file was processed with a MATLAB-based routine specifically designed to obtain the frequency decomposition of the noise signal. The frequency spectrum of the motor noise signal, *f*(*t*), was performed using the Fourier transform [22],

(1) $$F\left(j\cdot \omega \right)=\int_{0}^{\left(N-1\right)\cdot T}f\left(t\right)\cdot e^{-j\cdot \omega }\cdot dt$$

where ***F***(***j****·ω*) represents the function in the frequency domain related to the function in the time domain, *f*(*t*), *N* represents the number of samples through which the time signal has been acquired and *T* is the uniform sampling period. In this work, the fast Fourier transform (FFT) algorithm was used, as this method takes advantage of the periodicity and symmetry in the calculation of the discrete Fourier transform and has a low computational cost [23].

After performing the spectral analysis of the motor noise, the tailor-made speed estimation routine determines the fundamental rotor shaft mechanical frequency, *ω_F_* (Hz or revolutions per second), from some selected frequencies of the spectrum. Finally, the corresponding rotor speed, *Ω* (rad/s), is determined as:

(2) $$\Omega =2\cdot \pi \cdot \omega_{F}$$

Comparing the noise-estimated speed, *Ω*, with the actual value obtained with tachometers, *Ω_m_*, the relative value of the error speed is determined as:

(3) $$\varepsilon =\left|\frac{\Omega -\Omega_{m}}{\Omega_{m}}\right|$$

The standard deviation, *σ*, was used as a measure of the dispersion of the results. It is determined as:

(4) $$\sigma =\sqrt{\frac{1}{N}\cdot \sum_{i=1}^{N}{\left(x_{i}-\mu \right)}^{2}}$$

where *N* represents the number of recordings, *x_i_* represents the value of the *i*th recording in the data set and *μ* is the mean value of the data set. 

**Figure 2:**
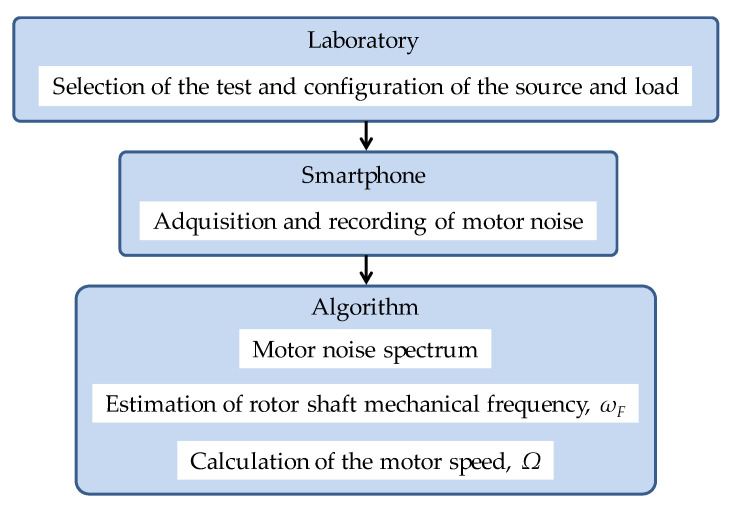
General scheme of the procedure followed to estimate the rotor speed.

The flowchart sketched in Figure 2 summarizes the general procedure followed to estimate the rotor speed in experimental tests.

**Figure 3:**
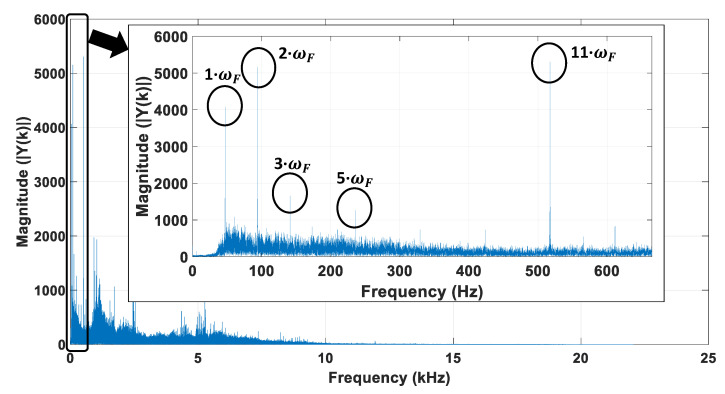
Frequency spectrum of the motor noise signal for no-load at 2820 r/min (295.3097 rad/s).

As an example, Figure 3 shows the frequency spectrum of the noise signal for a no-load motor feed at rated voltage at a measured speed of *Ω_m_* = 2820 r/min = 295.3097 rad/s (tachometers). It was obtained after applying the FFT transform to the corresponding audio file, the first step of the noise-estimation speed routine. The smartphone T1 with app ‘App1’ (Appendix A) was used to acquire and record a 30 s length noise signal with a sampling frequency of 44 kHz, well above the expected Shannon limit. 

As can be seen in Figure 3, the amplitudes corresponding to five frequencies (in ascending order), *ω*_1_ = *ω_F_*, *ω*_2_ = 2·*ω_F_*, *ω*_3_ = 3·*ω_F_*, *ω*_5_ = 5·*ω_F_*, and *ω*_11_ = 11·*ω_F_*, corresponding to the harmonics 1, 2, 3, 5, and 11, approximately, clearly stand out. The lowest peak frequency, that is, the fundamental frequency of the noise signal, *ω_F_* = 47.0618 Hz, should correspond to the rotor shaft mechanical rotation frequency. Then, the remaining frequencies should be related to the second, third, fifth, and 11th harmonics of the rotor shaft frequency. Table 1 shows the values of the frequencies corresponding to the five peaks of the magnitude of the spectrum of the noise signal. In this table, when the frequency of the first peak of amplitude is considered as the fundamental frequency, *ω_F_*_1_ = *ω*_1_/1 = 47.0618 Hz, the estimation of the rotor speed from that fundamental frequency, using (2), leads to *Ω* = 2·π·*ω*_1_ = 2·π·*ω_F_*_1_ = 2·π·47.0618 = 295.6980 rad/s = 2823.7080 r/min. Using (3), the relative error for the example shown is *ε* = 0.1315%, which is quite small. The preliminary results of this case demonstrate, pending further validation experiments, that the proposed method allows a proper rotor speed estimation.

Nevertheless, the remaining four peak frequencies, *ω_k_* = *k*·*ω_Fk_*, *k*∈{2,3,5,11}, could also have been considered to determine the fundamental (the mechanical shaft frequency) as *ω_Fk_* = *ω_k_*/*k* and therefore the speed:

(5) $${Ω}_{k}=Ω\left(\omega_{k}\right)=2\cdot \pi \cdot \omega_{Fk}=\left(2\cdot \pi \cdot \omega_{k}\right)/k$$

The third row of Table 1 shows the estimation of the fundamental frequency based on the five most significant harmonics of the spectrum of the motor noise, while the fourth and fifth rows show the corresponding values of the speed calculated by means of (5). Since there are five credible values of speed available, the estimation could be improved by using the mean value of the five estimated speed results, *Ω_mean_* = ∑*Ω_k_*/5 = 295.6674 rad/s = 2823.416 r/min. In this case, the relative error is reduced to *ε_mean_* = 0.1211%, which is smaller than that corresponding to the estimation based solely on the frequency of the first peak (7.9% error reduction). Nevertheless, in this case, the best estimation of the fundamental frequency and speed should have been based on the 11th harmonic. In this case the estimated speed result *Ω*_11_ = 295.5180 rad/s = 2821.989 r/min and the corresponding relative error would have been reduced to its minimum value, *ε_11_* = 0.0705%, as shown in the last row of Table 1. Although the speed can be estimated from any harmonic with a rather small error, this simple case was only intended to illustrate how a proper selection of the harmonic or harmonics used for calculating the rotor shaft frequency, *ω_F_*, can lead to a further improvement of the speed estimation. Section 2.1 shows the proposed method to improve the fundamental frequency and the corresponding speed estimations.

### 2.1. Speed Estimation Method

The analysis of about 700 motor-noise audio files, recorded with sampling frequencies from 8 kHz to 44 kHz, shows that when truncating the spectrum after the 11th harmonic, a sufficient number of frequency peaks are obtained to be able to properly identify the rotation frequency of the motor shaft (*ω_F_*) and, consequently, its speed (*Ω*). To optimize computing resources, the identification of the motor shaft rotation frequency (*ω_F_*) is performed by looking for the 11 largest frequency peaks below 665 Hz. That frequency limit is just 5 Hz above 660 Hz, the frequency corresponding to the 11th harmonic for 60 Hz motors, which also includes the case of 50 Hz motors.

**Figure 4:**
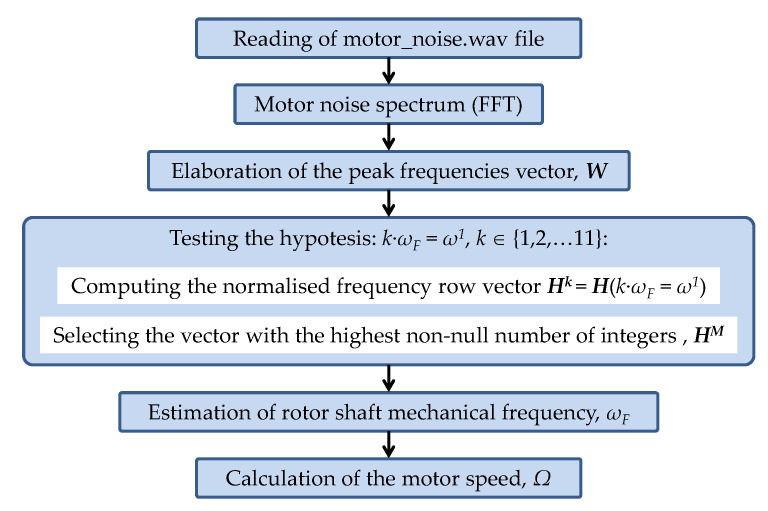
Flowchart of the speed estimation method.

The flowchart in Figure 4 (corresponding to the third block, ‘Algorithm’, in Figure 2) summarized the steps for computing the motor speed from the analysis of the recording of a motor-noise signal. For a better understanding, the procedure of analysis of a motor-noise recording will be described step-by-step by means of an example. The case shown corresponds to a two-pole induction motor at no-load, rotating at a measured speed (tachometer) of *ω_m_* = 2820 r/min, that is, with a shaft mechanical rotation frequency of *ω_F_* = 47 Hz.

First, the frequency spectrum is restricted from 0 Hz to 665 Hz (although it could have been limited to 555 Hz in this case). Then, the row vector of peak frequencies, ***W***, with the 11 most significant harmonics in magnitude is set:

(6) $$W=\left(\omega^{1},\omega^{2},\omega^{3},\omega^{4},\omega^{5},\omega^{6},\omega^{7},\omega^{8},\omega^{9},\omega^{10},\omega^{11}\right)$$

**Figure 5:**
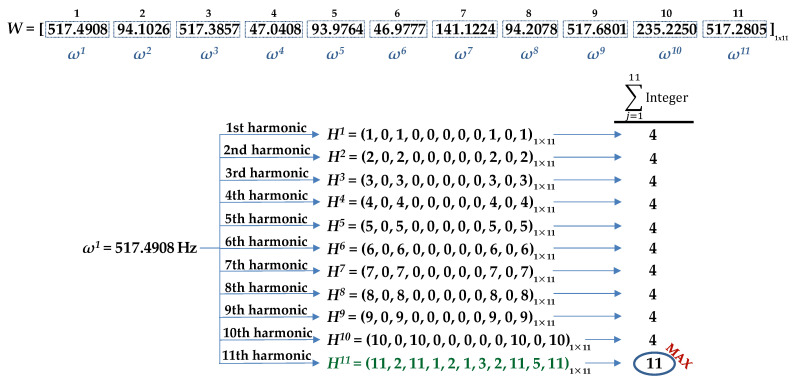
Row vector of peak frequencies, ***W***, normalized frequency row vectors, ***H^k^*** and counting of the non-null elements obtained for all possible harmonic orders of the first element, *ω*^1^.

The peak frequencies of the spectrum in decreasing order of magnitude, *ω^n^*, components of ***W***, are shown in the upper part of Figure 5.

Once the vector of peak frequencies ***W*** have been identified, the algorithm proceeds to test the possibility that its first element, *ω*^1^ (517.4908 Hz), the frequency with the larger amplitude, could correspond to the first, second, …, or to the 11th harmonic of the shaft rotation frequency, *ω_F_*. That is, it tests the possibility that *k*·*ω_F_* = *ω*^1^ = 517.4908 Hz, with *k* ∈ {1,2,…11}. With that purpose, first, a draft version of the normalized frequency row vector ***H*^1^** = ***H***(1·*ω_F_* = *ω*^1^) is computed. The elements of ***H*^1^** = (*h_n_*^1^) = (1·*ω^n^*/*ω*^1^) are the normalized frequency (possible harmonic orders) of the considered case (1·*ω^n^*/*ω*^1^). They are computed with three decimal digits.

(7) $$H^{1}=H\left(\omega_{F}=\omega^{1}\right)=\left(1\cdot \frac{\omega^{n}}{517.4908}\right)=\left(1.000\;\;0.182\;\;1.000\;\;0.091\;\;0.182\;\;0.091\;\;0.273\;\;0.182\;\;1.000\;\;0.455\;\;1.000\right)\;$$

After the draft version of the row vector ***H*^1^** has been computed, the elements *h_n_*^1^ are rounded to the nearest integer when the difference between the frequency ratio, *h_n_*^1^ and the nearest integer is lower than 10^−3^. In any other case, the element is discarded and replaced by 0.

(8) $$H^{1}=H\left(\omega_{F}=\omega^{1}\right)=\left(1\;\;0\;\;1\;\;0\;\;0\;\;0\;\;0\;\;0\;\;1\;\;0\;\;1\right)$$

In this case, the vector ***H*^1^** has four non-null elements and seven null elements, as can be seen in Figure 5. This means that the hypothesis *ω_F_* = *ω*^1^ leads to the identification of only four harmonics (non-null elements), all of them identified as the first (fundamental) harmonic of the shaft rotation frequency.

Similarly, the remainder of the row vectors are obtained, as shown in Figure 5:

(9) $$H^{2}=H\left(2\cdot \omega_{F}=\omega^{1}\right)=\left(2\cdot \left(\omega^{n}/517.4908\right)\right)\;H^{3}=H\left(3\cdot \omega_{F}=\omega^{1}\right)=\left(3\cdot \left(\omega^{n}/517.4908\right)\right)\;\ldots \;H^{k}=H\left(k\cdot \omega_{F}=\omega^{1}\right)=\left(k\cdot \left(\omega^{n}/517.4908\right)\right)\;\ldots \;H^{11}=H\left(11\cdot \omega_{F}=\omega^{1}\right)=\left(11\cdot \left(\omega^{n}/517.4908\right)\right)$$

Then, the number of integer ratios, or possible harmonics, for each considered case are quantified (Figure 5). Finally, the row vector, ***H^M^*** *=* (*h_n_^M^*), which has the highest non-null number of integers (or the highest number of possible harmonics of the considered case), is chosen as the best candidate for describing the proper harmonic order, ***M***, of each element of the frequency vector, ***W*** = (*ω^n^*). This finally leads to the best identification of the rotor shaft frequency, *ω_F_*, as the average ratio of each non-null component divided by its respective order:

(10) $$\omega_{F}=\left(\sum^{\;}(\omega^{n}/h_{n}^{M}\right))/n$$

For the example in Figure 5, ***H*^11^** is the vector with the highest number of integer ratios or possible harmonic coincidence. In other words, the highest number of harmonic identifications occurs under the hypothesis that the highest amplitude frequency, *ω*^1^ = 517.4908 Hz, represents the 11th harmonic.

(11) $$H^{11}=\left(11,\;2,\;11,\;1,\;2,\;1,\;3,\;2,\;11,\;5,\;11\right)=\left(h_{1}^{11},h_{2}^{11},h_{3}^{11},h_{4}^{11},h_{5}^{11},h_{6}^{11},h_{7}^{11},h_{8}^{11},h_{9}^{11},h_{10}^{11},h_{11}^{11},\right)$$

It is interesting to observe that, in this case, under that hypothesis, five different harmonics (1, 2, 3, 4, 5, and 11) of the rotor shaft frequency are identified. Each element of ***H*^11^** leads to a likely identification of the rotor shaft frequency

(12) $$\omega_{Fn}^{11}=(\omega^{n}/h_{n}^{11}),\;n\in \left\{1,\;2,\;\ldots ,\;11\right\}$$

and rotor speed,

(13) $$\Omega_{n}^{11}=2\cdot \pi \cdot \omega_{Fn}^{11}=2\cdot \pi \cdot \left(\omega^{n}/h_{n}^{11}\right),\;n\in \left\{1,\;2,\;\ldots ,\;11\right\}$$

Finally, the estimation of the rotor shaft (fundamental) frequency, *ω_F_*, is obtained as the average ratio of each component divided by its respective order (10). In this case, the result is:

(14) $$\omega_{F}=\frac{1}{11}\overset{11}{\underset{n=1}{\sum }}\Omega_{n}^{11}=\frac{1}{11}\overset{11}{\underset{n=1}{\sum }}\frac{\omega^{n}}{h_{n}^{11}}=47.0377\;\mathrm{Hz}$$

Thus, the estimation of the motor speed is calculated using (2), resulting in *Ω* = 2822.3 r/min.

## 3. Results

This section displays the experimental validation of the algorithm proposed in the previous section. Thus, the accuracy and the robustness of the method is analysed under different conditions. It is important to highlight that the laboratory where the tests were carried out is not soundproof, which means that the background noise in the audio recordings presents is an important challenge.

Four types of tests were performed on the induction motor, combining:

- The feeding source
  ○ The (50 Hz) network through a variable ratio transformer, which allows the adjusting of the supply voltage
  ○ An inverter or frequency converter, which enables adjustments to be made to both the voltage and frequency supplied to the motor.
- The mechanical load
  ○ No mechanical load: the motor shaft is free
  ○ Direct current generator, which feeds a variable resistive load, allowing for changes to be made to the motor torque.

Table 2 summarizes the range of parameters used along the experiments.

The influence of each parameter and range of values in the frequency spectrum and in the calculation of the speed is analyzed. In all cases, very good estimations are obtained. The results presented correspond to the recordings obtained with a length, *t* = 30 s, sampling frequency, *F_sampling_* = 44 kHz, recording application App1, smartphone T1 and distance *m* = 5 cm. The main technical characteristics of the induction motors and devices used in this work are provided in Appendix A.

### 3.1. Motor Feeding from the Grid through a Variable Ratio Transformer without Mechanical Load

The objective in this section is to validate that the algorithm is able to properly estimate the speed of an induction motor feeding from the grid when rotated freely, without any mechanical load coupled to its shaft. The measurement was made on the rotor speed of a 50 Hz, two-pole induction motor, fed from the 50 Hz grid by means of a variable ratio transformer, or variable ratio transformer which allows for the adjusting of the rotor speed from 2820 r/min to 3000 r/min, or the rotor shaft mechanical frequency from 47 Hz to 50 Hz. 

**Figure 6:**
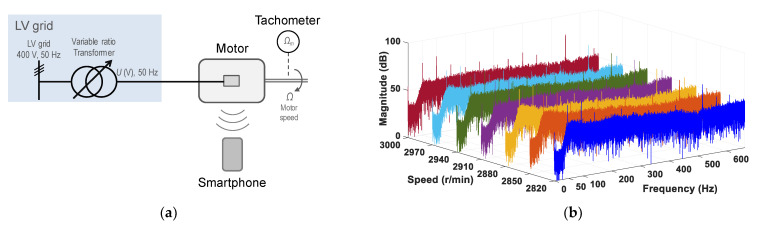
No load coupled to the motor shaft: (**a**) the test configuration; (**b**) the spectra of the motor noise signals.

Figure 6a shows a sketch of the test configuration; Figure 6b shows the spectra of the motor noise signals. As can be seen, the spectrum of each rotor speed shows peak frequencies around 550 Hz (11th harmonic), around 50 Hz (fundamental or first harmonic) and 100 Hz (second harmonic), with considerably higher magnitude than the remainder of the frequencies of the spectrum. 

Table 3 and Table 4 summarize the results. For each rotor actual speed *Ω_m_* (average value of the values measured with two tachometers), Table 3 shows, in the second column, the corresponding rotor shaft mechanical frequency, *ω_m_*, using (2) followed by the values of the frequencies corresponding to the 11 peaks of the magnitude of the spectrum of the noise signal. Table 4 shows the estimated values of rotor shaft mechanical frequency, *ω_F_*, and rotor speed, *Ω*, corresponding to each actual speed, *Ω_m_*, determined by means of the proposed method. As can be seen, the rotor speed is always well-estimated. The relative error is very small, since for the worst case, it is under 0.08%. The standard deviation of the relative error in the estimation of the speed from the individual peak frequencies is also very small, always under 0.03 Hz (fifteen audio recordings were considered for the calculation of the standard deviation in each case). 

### 3.2. Motor Feeding from the Grid through a Variable Ratio Transformer with a DC Generator as Mechanical Load

**Figure 7:**
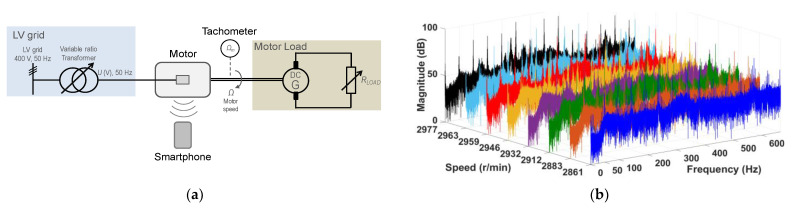
DC generator coupled to the motor shaft: (**a**) the test configuration; (**b**) the spectra of the motor noise signals.

In this test battery, the motor was fed from the grid through a variable ratio transformer while a DC generator is coupled to its shaft. A variable resistance, fed by the DC generator, allows the adjusting of the mechanical load and, accordingly, the rotor speed of the motor. For that purpose, with that configuration of source and mechanical load, the measurements were made on the rotor speed of a 50 Hz, two-pole induction motor, fed from the 50 Hz grid by means of a variable ratio transformer and varying the DC generator variable resistance load, which allows for the adjusting of the rotor speed from 2861 r/min to 2977 r/min, or the rotor shaft mechanical frequency from 47.68 Hz to 49.62 Hz. Figure 7a shows a sketch of the test configuration; Figure 7b shows the spectra of the motor noise signals. A simple visual comparison of the frequency spectrum of the motor load test of Figure 7b with that corresponding to the no mechanical load of Figure 6b shows that the presence of the DC generator in the load test increases complexity and level of harmonics of the noise spectra.

Table 5 and Table 6 summarize the results. The results corresponding to values R0–R7 of the load resistance correspond to the motor feed at rated voltage. For each rotor actual speed (average value of the values measured with two tachometers), Table 5 shows the corresponding rotor shaft mechanical frequency using (2) in the third column followed by the values of the frequencies corresponding to the 11 peaks of the magnitude of the spectrum of the noise signal. 

Table 6 shows the estimated values of rotor shaft mechanical frequency, *ω_F_*, and rotor speed, *Ω*, corresponding to each actual speed, *Ω_m_*, determined by means of the proposed method. As can be seen, the rotor speed is always well-estimated. The relative speed error is very small, since for the worst case (rated voltage), it is under 0.1 %, only slightly exceeded (0.126 %) for the case R7*, when the motor is fed with a voltage under its rated value. The standard deviation of the relative error in the estimation of the speed from the individual peak frequencies is also very small, always under 0.02 Hz (three audio recordings were considered for the calculation of the standard deviation in each case).

As shown in Table 6, the case R0 corresponds to the case with lower torque. In case R7, two additional exceptional situations were studied: the motor experiences a reduction of the voltage in relation to nominal voltage (R7*) and the opposite case, when the motor experiences an overvoltage in its point of connection (R7**). Regarding the reduced values of the errors (*ε* ≤ 0.126 %) and standard deviation obtained (*σ* ≤ 0.02 Hz), it can be concluded that the performance of the algorithm is not affected by coupling a direct current generator as a mechanical load. 

### 3.3. Motor Feeding with a Frequency Converter without Mechanical Load

The objective in this section is to validate that the algorithm is able to properly estimate the speed of an induction motor feeding from a frequency converter when rotated freely, without any mechanical load coupled to its shaft. The measurement was made on the rotor speed of a 50 Hz, two-pole induction motor, fed from an inverter at line frequencies from 20 Hz to 60 Hz, which allows for the adjusting of the rotor speed from 1200 r/min to 3600 r/min, approximately (or rotor shaft mechanical frequencies from 20 Hz to 60 Hz). 

**Figure 8:**
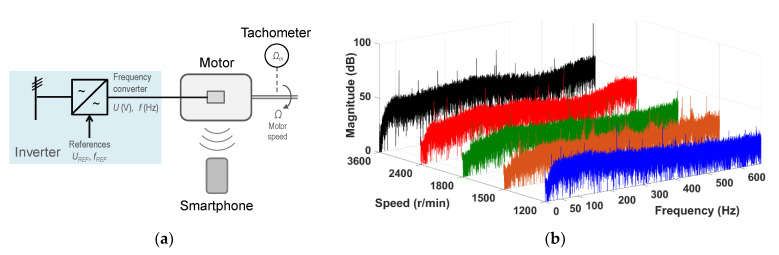
No load coupled to the motor shaft: (**a**) the test configuration; (**b**) the spectra of the motor noise signals.

Figure 8a shows a sketch of the test configuration; Figure 8b shows the spectra of the motor noise signals. Figure 8b shows that the higher the inverter frequency, the higher the harmonic content of the spectra.

Table 7 and Table 8 summarize the results. For each rotor actual speed (average value of the values measured with two tachometers), *Ω_m_*, Table 7 shows, in the second column, the corresponding rotor shaft mechanical frequency, *ω_m_*, using (2) followed by the values of the frequencies corresponding to the 11 peaks of the magnitude of the spectrum of the noise signal. Table 8 shows the estimated values of rotor shaft mechanical frequency, *ω_F_*, and rotor speed, *Ω*, corresponding to each actual speed, *Ω_m_*, determined by means of the proposed method. As can be seen, the rotor speed is always well-estimated. The relative error is very small, since for the worst case, it is under 0.26%. The standard deviation of the relative error in the estimation of the speed from the individual peak frequencies is also very small, always under 0.04 Hz (five audio recordings were considered for the calculation of the standard deviation in each case).

As shown in Table 8, the reduced errors prove the robustness of the proposed method. It can be concluded that the performance of the algorithm is not affected by feeding the motor through a frequency converter instead of directly from the grid. 

### 3.4. Motor Feeding with a Frequency Converter with a DC Generator as Mechanical Load

**Figure 9:**
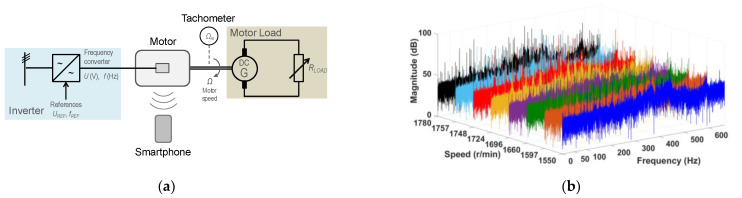
DC generator coupled to the motor shaft: (**a**) the test configuration; (**b**) the spectra of the motor noise signals.

In this test battery, the motor was fed from an inverter or frequency converter while a DC generator is coupled to its shaft, as a mechanical load. The frequency and voltage settings of the converter allows for setting the synchronous speed of the induction motor. A variable resistance, fed by the DC generator, allows for the adjusting of the mechanical load and, accordingly, the actual rotor speed of the motor. With that configuration of source and mechanical load, the rotor speed of a 50 Hz, two-pole induction motor was measured, feeding from the converter with variable frequencies (and voltages) from 10 Hz to 60 Hz, approximately and modifying the DC generator variable resistance load which allows for the adjusting of the actual rotor speed from 2861 r/min to 2977 r/min, or the rotor shaft mechanical frequency from 47.68 Hz to 49.62 Hz. Figure 9a shows a sketch of the test configuration; Figure 9b shows the spectra of the motor noise signals. A simple visual comparison of the frequency spectrum of the motor load with variable frequency test of Figure 9b, with the corresponding to the motor fed from the grid of Figure 7b, shows that even though the magnitudes of the harmonic are lower in the case of working at a reduced speed (frequency converter), the harmonic content of the spectra in Figure 9b is considerably higher.

Table 9 shows the values of measured rotor speeds for each considered converter frequency and load resistance (mechanical load of the motor). For each converter frequency, as the mechanical load increases, the rotor speed decreases, as expected. Table 10, Table 11 and Table 12 summarize the results of the load tests corresponding to a frequency converter of 10 Hz, 20 Hz, and 30 Hz, respectively. Table 10, Table 11 and Table 12 show, for each converter frequency, the measured motor speed, *Ω_m_*, and rotor shaft mechanical frequency, *ω_F_*, as well as the estimated values of rotor shaft mechanical frequency, *ω_F_*, and rotor speed, *Ω*, corresponding to each actual speed, *Ω_m_*, determined by means of the proposed method. The rotor speed is always properly estimated, with a reduced error. The relative speed error is very small, since for the worst case (rated voltage), it is 0.0659 %. The standard deviation of the relative error in the estimation of the speed from the individual peak frequencies is also very small, always under 0.02 Hz (three audio recordings were considered for the calculation of the standard deviation in each case).

### 3.5. Other Tests

In order to test the robustness of the proposed method, the experimental section was extended with some complementary tests, including situations with two induction motors, motors with more than two poles and different distances between the motor and the smartphone. 

#### 3.5.1. Four-Pole Motor

**Figure 10:**
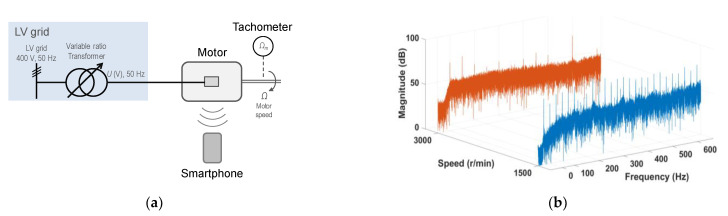
No load coupled to the motor shaft: (**a**) the test configuration; (**b**) the spectra of the motor noise signals.

This subsection shows the results of the analysis of the audio recordings of a 50 Hz, four-pole induction motor whose shaft can rotate freely (no mechanical load). The motor was supplied from the grid by means of a variable ratio transformer. After adjusting the supply voltage, the actual measured speed is, according to the tachometer, *Ω_m_* = 1500 r/min, and the corresponding rotor shaft mechanical frequency using (2), *ω_m_* = 25 Hz. Figure 10a sketches the test configuration; Figure 10b shows a comparison between the frequency spectrum of the four-pole motor (1500 r/min) and a two-pole motor (3000 r/min).

As shown in Figure 10b, a higher number of intense harmonics are observed in the spectrum of the four-pole motor. Table 13 shows the first 11-highest harmonic peaks and the corresponding harmonic order considering the highest amplitude frequency, *ω*^1^, corresponds to the ninth harmonic, that is, the components of vectors **W** and ***H*^9^** *= **H***(*9·ω_F_* = *ω*^1^). 

The estimation of the fundamental mechanical frequency of the rotor shaft for this audio leads to *ω_F_* = 24.98 Hz, which corresponds to an estimated rotor speed of *Ω* = 1498.8 r/min, that is, the error in the estimation is ε = 0.0816 %.

Table 14 shows the results corresponding to the mechanical rotor frequency and rotor speed estimations when the distance between the smartphone and the motor is increased from 5 cm to 150 cm.

Five audio recordings were considered for the calculation of the standard deviation in each case. As shown in Table 14, the frequency estimations are quite similar to the measured frequency, *ω_F_* ≈ *ω_m_*, regardless of the distance, demonstrating the good performance of the proposed method.

#### 3.5.2. Two Motors

**Figure 11:**
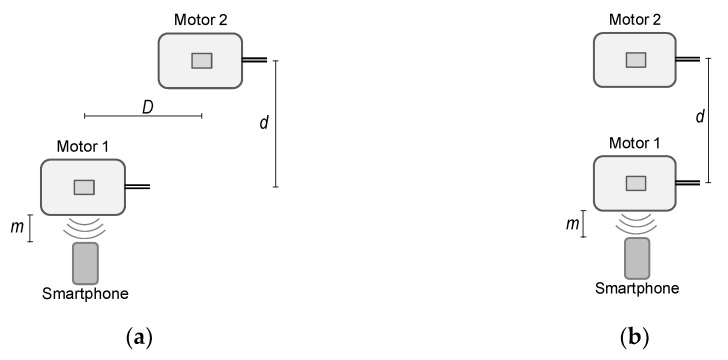
No load coupled to the motor shaft: (**a**) separated motors; (**b**) faced motors.

The purpose of this subsection is to validate that the algorithm can estimate the speed of the motor when there is another similar motor operating simultaneously at a different speed in the neighborhood. Figure 11 shows the layout of the two-motor set-up considered: (a) separated motors and (b) faced motors.

Motor 1 is the *reference motor*, that is, the motor whose speed is to be estimated. The noise from Motor 2, the *background motor*, should be discarded as background noise. The values of the distances are *d* = 1 m, *D* = 1 m and the distance of the smartphone, *m* = 5 cm. Table 15 shows the results corresponding to the considered speeds of both motors (averaged values), the reference motor, *Ω_m_*_1_ = *Ω_m_*, and the background motor, *Ω_m_*_2_. The estimated rotor frequencies and speeds, as well as the absolute relative errors, are also presented.

As shown in Table 15, the algorithm leads to a good estimation of the reference motor speed, regardless of whether the *reference motor* speed is higher than, lower than or similar to the *background motor* speed. The relative error is always less than 0.11 %.

The impact of the distance between the smartphone and the *reference motor* in the performance of the algorithm has also been analyzed. Table 16 shows that the results of the *reference motor* speed is *Ω_m_*_1_ = 2880 r/min while the *background motor* speed is *Ω_m_*_2_ = 2940 r/min.

Three audio recordings were considered for the calculation of the standard deviation in each case. As shown in Table 16, the proposed algorithm is able to determine the rotor speed regardless of the distance between the motor and the smartphone.

## 4. Discussion

As explained in Section 2, the proposed method is based on the hypothesis that the higher peak frequency of the noise signal spectrum, *ω*^1^, corresponds to one of the first eleven harmonics, *k*, of the rotor shaft mechanical frequency, ***H^k^*** *= **H***(*k·ω_F_ = ω*^1^). Once it has determined the proper order of the harmonic, *k*, as corresponding to the vector ***H^k^*** with the higher number of non-null elements, the algorithm estimates the fundamental rotor shaft mechanical frequency using (2) and, finally, the motor speed using (1). 

It is important to highlight that the frequency band of smartphone microphones are optimized for voice applications (20 Hz–20 kHz, approximately). As a result, the measurement of motor speeds below the 20 Hz limit might be outside the scope of the proposed method. For these low frequencies, if the method were exclusively dependent on detecting the first harmonic, it might not be suitable for speed measurement lower than 20 Hz. However, the proposed method can overcome this drawback because it is not based only on the first (fundamental) harmonic of the noise spectrum. The experimental results have shown that the method leads to a correct motor speed determination with frequencies as low as 10 Hz. The proposed method uses a set of harmonics (frequency peaks) between the first and the 11th (in the basic version of the algorithm) to determine the motor speed. This distinctive feature is successful despite the limited smartphone microphone bandwidth. The method is fully capable of measuring motor speeds with low frequency, since their multiples (higher harmonics) of the fundamental can be found, leading to the determination of the motor speed.

In a very small percentage of the over 700 motor noise signal files analyzed (less than 2.5%), all corresponding to the four-pole motor, the higher peak frequency of the noise signal spectrum, *ω*^1^, corresponds to one harmonic higher that the 11th. In these cases, the main hypothesis of the proposed method was not fulfilled and, as a result, the rotor speed cannot be properly estimated. Although the proposed method is able to achieve a proper estimation of the motor speed, in order to deal with these exceptional cases, an extension of the method is proposed. The extension of the method only requires the capture of a higher number of peak frequencies of the noise spectrum and the testing of more frequency peaks as possible harmonics of the rotor shaft mechanical frequency. 

### An Extended Method

In the extended method, the frequency vector ***W*** is extended to capture the 120 first frequency peaks of the motor noise signal spectrum. In other words, the number of elements of the frequency vector ***W*** is raised from 11 to 120 elements. Then, the first five higher peak frequencies of the spectrum, (*ω*^1^, *ω*^2^, *ω*^3^, *ω*^4^, *ω*^5^), are selected and it is checked whether each of these five elements may fit between the first harmonic of the rotating shaft frequency and the 120th harmonic. Specifically, the harmonic vector with 11 elements, ***H***, is now enlarged to five harmonic vectors of 120 elements. The extended algorithm sequentially computes five estimations of the rotor shaft mechanical frequency, exploring the five different hypotheses: *ω_F_*^1^, *ω_F_*^2^, *ω_F_*^3^, *ω_F_*^4^, *ω_F_*^5^. When a frequency estimation, *ω_F_^i^*, significantly deviates from the remainder values, it is discarded, and the rotor shaft mechanical frequency is estimated by averaging the remainder frequency estimations. Table 17 summarizes the main parameters of the proposed and extended versions of the method. It is important to note that the execution time of the extended method is only 55 ms (13 %) longer than the original version.

Although all the noise motor noise records analyzed in this work have led to a proper motor speed estimation, if it were necessary, the extended method could be further extended including more than 120 peak frequencies in ***W*** or checking more than the first five higher peak frequencies of the spectrum to estimate the motor speed. 

The estimated speed with the proposed method is 2822.3 r/min and with the extended method is 2821.9 r/min. Thus, the algorithm leads to a good estimation of the motor speed, regardless the selected method.

## 5. Conclusions

The present work has proposed a simple, low-cost, non-intrusive method for the determination of the motor speed, based on a simple smartphone as an acquisition and recording device, and a MATLAB routine as a rotor speed estimation algorithm. The proposed method of motor speed estimation is based on the analysis of the frequency spectrum of the motor noise record. It has been experimentally tested in the laboratory for different configurations of the power supply of the motor (variable ratio transformer or frequency converter) and mechanical loads (DC generator and no mechanical load). A broad battery of tests has been used in the experimental section of the work, including two different smartphones placed at different distances from the tested motor, using two different recording apps, with two-pole and four-pole induction motors.

The values of the rotor speed estimation reported in Section 3 are in full agreement with the experimental tests results, which proves the availability, versatility, robustness, and accuracy of the proposed method. The relative errors on the motor speed estimation are always lower than 0.151% in the worst-analyzed case. Accordingly, the methodology based on the analysis of frequency spectrum of motor noise can be considered a suitable new tool to be added to the catalogue of techniques to estimate mechanical rotor speed. 

The authors are working on the developing of an app to integrate the recording of the motor noise and the algorithm of motor speed calculation, as well as towards the development of motors noise-based diagnostic tools.

## Figures and Tables

**Table 1:** Five frequency peaks of the spectrum corresponding to the motor noise signal for no-load test at 2820 r/min (295.3097 rad/s).

Order	First-Fundamental *k* = 1	Second *k* = 2	Third *k* = 3	Fifth *k* = 5	Eleventh *k* = 11
Harmonic	*ω*_1_ = *ω_F_*_1_	*ω*_2_ = 2·ω*_F_*_2_	*ω*_3_ = 3·ω*_F_*_3_	*ω*_5_ = 5·ω*_F_*_5_	*ω*_11_ = 11·ω*_F_*_11_
Frequency (Hz)	47.0618	94.1237	141.1855	235.3302	517.3647
Fundamental (Hz)	47.0618	47.0619	47.0618	47.0660	47.0332
Speed (rad/s)	295.6980	295.6983	295.6982	295.7247	295.5180
Speed (r/min)	2823.708	2823.711	2823.710	2823.962	2821.989
Relative error (%)	0.1315	0.1316	0.1316	0.1405	0.0705

**Table 2:** Range of values selected for the parameters along the tests.

Parameters	Range of Values
Recording time, *t* (s)	[5, 10, 20, 30]
Sampling frequency, *F_sampling_* (kHz)	[8, 16, 44]
Distance between the mobile phone and the motor, *m* (cm)	[5, 25, 50, 75, 100, 150]
Mobile application for recording	App1, App2
Smartphone	T1, T2

**Table 3:** No load coupled to the motor shaft: the rotor shaft mechanical frequency and the values of the frequencies corresponding to the 11 peaks of the magnitude of the spectrum of the noise signal.

Speed (r/min)	Frequency (Hz)											
*Ω_m_*	*ω_m_*	*ω* ^1^	*ω* ^2^	*ω* ^3^	*ω* ^4^	*ω* ^5^	*ω* ^6^	*ω* ^7^	*ω* ^8^	*ω* ^9^	*ω* ^10^	*ω* ^11^
2820	47.00	517.49	94.10	517.39	47.04	93.98	46.98	141.12	94.21	517.68	235.23	517.28
2850	47.50	47.50	95.01	522.66	95.07	522.77	47.57	522.81	95.11	142.53	237.54	94.88
2880	48.00	96.00	47.99	528.05	143.98	527.88	95.85	192.01	240.02	191.95	239.94	96.10
2910	48.50	97.09	48.53	533.96	97.03	194.16	533.85	145.62	97.15	48.49	533.81	242.69
2940	49.00	539.34	49.04	539.47	98.06	245.15	539.51	147.09	539.23	245.21	539.55	294.19
2970	49.50	99.07	49.52	544.95	148.63	544.43	49.48	247.69	544.30	247.61	544.39	49.42
3000	50.00	549.54	49.96	99.93	400.68	549.41	249.80	549.75	149.87	50.01	449.63	199.83

**Table 4:** No load coupled to the motor shaft: estimated speeds and frequencies, relative errors, and standard deviations.

Experimental		Algorithm		Relative Error	Standard Deviation
*Ω_m_* (r/min)	*ω_m_* (Hz)	*Ω* (r/min)	*ω_F_* (Hz)	*ε* (%)	*σ* (Hz)
2820	47.00	2822.3	47.04	0.0800	0.0166
2850	47.50	2851.0	47.52	0.0340	0.0205
2880	48.00	2879.6	47.99	0.0140	0.0292
2910	48.50	2912.1	48.53	0.0710	0.0114
2940	49.00	2942.1	49.04	0.0730	0.0135
2970	49.50	2970.7	49.51	0.0250	0.0117
3000	50.00	2997.9	49.97	0.0700	0.0117

**Table 5:** DC generator coupled to the motor shaft: rotor shaft mechanical frequency and values of the frequencies corresponding to the 11 peaks of the magnitude of the spectrum of the noise signal.

Resistance	Speed (r/min)	Frequency (Hz)											
	*Ω_m_*	*ω_m_*	*ω* ^1^	*ω* ^2^	*ω* ^3^	*ω* ^4^	*ω* ^5^	*ω* ^6^	*ω* ^7^	*ω* ^8^	*ω* ^9^	*ω* ^10^	*ω* ^11^
R7	2861	47.68	238.27	142.95	238.32	333.58	190.62	571.83	333.62	571.89	381.23	95.30	285.93
R6	2883	48.05	240.31	384.51	144.19	336.44	576.77	384.46	240.27	336.39	576.71	96.12	432.58
R5	2912	48.53	242.67	339.74	145.60	339.69	242.71	582.45	388.31	582.36	194.14	388.23	97.07
R4	2932	48.87	244.25	341.95	146.55	586.19	195.40	342.01	244.18	390.79	586.23	439.64	342.07
R3	2946	49.10	245.57	343.80	147.35	392.92	343.86	98.22	589.39	342.03	245.66	540.27	589.43
R2	2959	49.32	246.62	345.27	246.561	345.203	394.584	147.967	394.547	98.65	591.89	346.61	246.69
R1	2963	49.38	246.96	395.13	345.73	148.17	592.69	230.75	345.69	246.90	98.77	592.65	230.03
R0	2977	49.62	248.09	347.33	396.96	230.83	396.89	347.29	148.86	99.23	396.85	198.47	230.56

**Table 6:** DC generator coupled to the motor shaft: estimated speeds and frequencies, relative errors, and standard deviations.

Resistance	Experimental		Algorithm		Relative Error	Standard Deviation
	*Ω_m_* (r/min)	*ω_m_* (Hz)	*Ω* (r/min)	*ω_F_* (Hz)	*ε* (%)	*σ* (Hz)
R7	2861	47.68	2859.3	47.66	0.0594	0.0097
R7 *	2820	47.00	2816.5	46.94	0.1255	0.0091
R7 **	2860	47.67	2860.4	47.67	0.0133	0.0090
R6	2883	48.05	2883.7	48.06	0.0225	0.0154
R5	2912	48.53	2912.0	48.53	0.0014	0.0050
R4	2932	48.87	2931.1	48.85	0.0323	0.0045
R3	2946	49.10	2947.0	49.12	0.0354	0.0044
R2	2959	49.32	2959.3	49.32	0.0102	0.0055
R1	2963	49.38	2963.3	49.39	0.0086	0.0193
R0	2977	49.62	2976.9	49.62	0.0021	0.0047

* DC load resistance adjusted at R7 value but the feeding voltage was slightly reduced under its rated value. ** DC load resistance adjusted at R7 value but the feeding voltage was slightly increased over its rated value.

**Table 7:** No load coupled to the motor shaft: the rotor shaft mechanical frequency and the values of the frequencies corresponding to the 11 peaks of the magnitude of the spectrum of the noise signal.

Speed (r/min)	Frequency (Hz)											
*Ω_m_*	*ω_m_*	*ω* ^1^	*ω* ^2^	*ω* ^3^	*ω* ^4^	*ω* ^5^	*ω* ^6^	*ω* ^7^	*ω* ^8^	*ω* ^9^	*ω* ^10^	*ω* ^11^
1200	20.00	220.34	360.55	220.25	99.97	493.92	50.11	492.00	220.19	440.67	100.16	360.62
1500	25.00	275.20	550.42	575.45	250.20	375.27	625.47	450.35	275.10	575.68	99.99	47.63
1800	30.00	330.29	150.14	330.21	30.03	47.50	555.87	147.33	330.61	60.16	391.03	349.77
2400	40.00	200.13	440.30	80.22	40.02	393.07	361.00	120.07	622.42	280.21	240.17	360.26
3600	60.00	659.81	59.97	299.91	659.73	119.97	659.96	179.94	120.43	539.84	660.15	50.01

**Table 8:** No load coupled to the motor shaft: estimated speeds and frequencies, relative errors, and standard deviations for no-load tests with frequency converter.

Experimental		Algorithm		Relative Error	Standard Deviation
*Ω_m_* (r/min)	*ω_m_* (Hz)	*Ω* (r/min)	*ω_F_* (Hz)	*ε* (%)	*σ* (Hz)
1200	20.00	1201.8	20.03	0.1510	0.0200
1500	25.00	1500.9	25.02	0.0620	0.0010
1800	30.00	1802.2	30.04	0.1230	0.0401
2400	40.00	2402.2	40.04	0.0912	0.0038
3600	60.00	3598.9	59.98	0.0300	0.0015

**Table 9:** DC generator coupled to the motor shaft: measured speed using the tachometer for different values of resistance.

Frequency Converter (Hz)	*Ω_m_* (r/min)	R7	R6	R5	R4	R3	R2	R1	R0
10.00	600	524.6	535	552.5	562.3	571	578	580	587.2
20.00	1200	1044	1070	1105	1128	1145	1159	1165	1179
25.00	1500	1301	1336	1384	1413	1435	1453	1460	1478
30.00	1800	1550	1597	1660	1696	1724	1748	1757	1780
40.00	2400	2031	2112	2185	2262	2301	2334	2345	2375
60.00	3600			2741	3163	3332	3425	3452	3519

**Table 10:** DC generator coupled to the motor shaft: estimated speeds and frequencies, relative errors, and standard deviations for load tests at 10 Hz.

Resistance	Experimental		Algorithm		Relative Error	Standard Deviation
	*Ω_m_* (r/min)	*ω_m_* (Hz)	*Ω* (r/min)	*ω_F_* (Hz)	*ε* (%)	*σ* (Hz)
R7	524.6	8.74	524.4	8.74	0.0301	0.0010
R6	535.0	8.92	535.3	8.92	0.0475	0.0006
R5	552.5	9.21	552.3	9.21	0.0405	0.0014
R4	562.3	9.37	562.5	9.37	0.0292	0.0005
R3	571.0	9.52	571.0	9.52	0.0035	0.0003
R2	578.0	9.63	578.3	9.64	0.0464	0.0005
R1	580.0	9.67	580.4	9.67	0.0676	0.0016
R0	587.2	9.79	587.4	9.79	0.0412	0.0012

**Table 11:** DC generator coupled to the motor shaft: estimated speeds and frequencies, relative errors, and standard deviations for load tests at 20 Hz.

Resistance	Experimental		Algorithm		Relative Error	Standard Deviation
	*Ω_m_* (r/min)	*ω_m_* (Hz)	*Ω* (r/min)	*ω_F_* (Hz)	*ε* (%)	*σ* (Hz)
R7	1044	17.40	1044.1	17.40	0.0098	0.0065
R6	1070	17.83	1069.5	17.83	0.0434	0.0032
R5	1105	18.42	1105.1	18.42	0.0116	0.0007
R4	1128	18.80	1127.7	18.80	0.0277	0.0031
R3	1145	19.08	1144.7	19.08	0.0248	0.0007
R2	1159	19.32	1159.8	19.33	0.0659	0.0001
R1	1165	19.42	1165.2	19.42	0.0161	0.0011
R0	1179	19.65	1179.1	19.65	0.0097	0.0022

**Table 12:** DC generator coupled to the motor shaft: estimated speeds and frequencies, the relative errors, and the standard deviations for load tests at 30 Hz.

Resistance	Experimental		Algorithm		Relative Error	Standard Deviation
	*Ω_m_* (r/min)	*ω_m_* (Hz)	*Ω* (r/min)	*ω_F_* (Hz)	*ε* (%)	*σ* (Hz)
R7	1550	25.83	1549.1	25.82	0.0586	0.0197
R6	1597	26.62	1596.6	26.61	0.0258	0.0127
R5	1660	27.67	1660.0	27.67	0.0027	0.0140
R4	1696	28.27	1696.1	28.27	0.0068	0.0041
R3	1724	28.73	1724.3	28.74	0.0176	0.0003
R2	1748	29.13	1748.3	29.14	0.0150	0.0012
R1	1757	28.28	1757.0	29.28	0.0022	0.0023
R0	1780	29.67	1779.7	29066	0.0181	0.0019

**Table 13:** No load coupled to the motor shaft: first 11-highest harmonics and their respective order.

**Frequency Vector, *W* (Hz)**										
***ω*^1^**	***ω*^2^**	***ω*^3^**	***ω*^4^**	***ω*^5^**	***ω*^6^**	***ω*^7^**	***ω*^8^**	***ω*^9^**	***ω*^10^**	***ω*^11^**
224.79	399.63	499.53	149.85	624.42	649.38	400.11	74.92	199.81	299.72	274.74
**Harmonic Order Vector, *H*^9^**										
***h*** **_1_** **^9^**	***h*** **_2_** **^9^**	***h*** **_3_** **^9^**	***h*** **_4_** **^9^**	***h*** **_5_** **^9^**	***h*** **_6_** **^9^**	***h*** **_7_** **^9^**	***h*** **_8_** **^9^**	***h*** **_9_** **^9^**	***h*** **_10_** **^9^**	***h*** **_11_** **^9^**
9	16	20	6	25	26	0	3	8	12	11

**Table 14:** No load coupled to the motor shaft: estimated speeds and frequencies, relative errors, and standard deviation for each test.

Distance (cm)	Experimental		Algorithm		Relative Error	Standard Deviation
	*Ω_m_* (r/min)	*ω_m_* (Hz)	*Ω* (r/min)	*ω_F_* (Hz)	*ε* (%)	*σ* (Hz)
5	1500	25.00	1498.8	24.98	0.0816	0.0037
50	1500	25.00	1498.5	24.98	0.0976	0.0031
150	1500	25.00	1499.3	24.99	0.0472	0.0098

**Table 15:** No load coupled to the motor shaft: estimation of the reference motor speed, the frequency, and the relative error obtained in each test.

Test	Experimental			Algorithm		Relative Error
	*Ω_m_*_1_ (r/min)	*Ω_m_*_2_ (r/min)	*ω_m_*_1_ (Hz)	*Ω*_1_ (r/min)	*ω_F_*_1_ (Hz)	*ε* (%)
1: Separated motors	2820	3000	47.00	2823.0	47.05	0.1053
2: Separated motors	3000	2820	50.00	2997.5	49.96	0.0818
3: Faced motors	2880	2940	48.00	2879.3	47.99	0.0229
4: Faced motors	2940	2880	49.00	2939.7	49.00	0.0092

**Table 16:** No load coupled to the motor shaft: estimation of the frequency, speed and the relative errors for faced motors.

Distance (cm)	Experimental			Algorithm		Relative Error	Standard Deviation
	*Ω_m_*_1_ (r/min)	*Ω_m_*_2_ (r/min)	*ω_m_*_1_ (Hz)	*Ω*_1_ (r/min)	*ω_F_*_1_ (Hz)	*ε* (%)	*σ* (Hz)
5	2880	2940	48.00	2879.3	47.99	0.0229	0.0069
50	2880	2940	48.00	2879.3	47.99	0.0256	0.0178
150	2880	2940	48.00	2881.0	48.02	0.0337	0.0101

**Table 17:** Parameters of the proposed method and the extended method.

Differences	Proposed Method (Explained in Section 2)	Extended Method
Frequency vector size	11 × 1	120 × 1
Assumption	*ω* ^1^	*ω*^1^, *ω*^2^, *ω*^3^, *ω*^4^, *ω*^5^
Harmonic order	1 to 11	1 to 120
Computer time (s) *	0.403	0.458
Estimated frequency (Hz)	47.0377	47.0320

* On a desktop PC with Intel^®^ Core™ i7-6700HQ CPU 2.60 GHz.

## Data Availability

The data used for the manuscript are available on request.

## Appendix A

Table A1, Table A2, Table A3 and Table A4 summarize the main rated characteristics of the induction motors, DC generator, frequency converter and tachometers, respectively, used in this work.

**Table A1:** Technical characteristics of the induction motor used in the tests.

Motor	Voltage (V)	Current (A)	Power (kW)	Power Factor	Speed (r/min)	Frequency (Hz)
DL1021 DeLorenzo (two poles)	220/380 (Δ/Y)	4.5/2.6 (Δ/Y)	1.1	0.85	2820	50
1LA3163-4AA20 Siemens (four poles)	220/380 (Δ/Y)	38/22 (Δ/Y)	11	0.84	1460	50

**Table A2:** Characteristic of the direct current generator used in the load tests.

DC Generator	Voltage (V)	Current (A)	Speed (r/min)
DL 1024R de Lorenzo	195	0.3	3000

**Table A3:** Characteristic of the frequency converter used in this work.

Frequency Converter	Power	Speed Range in Open-Loop Mode (Hz)	Voltage (V)
Telemecanique Altivar 71	0.75 kW	1–100	200–240

**Table A4:** Characteristic of the tachometers used in these tests.

Tachometer	Tac1	Tac2
Range	2.5 to 99999 r/min	5 to 99999 r/min
Precision	±0.051% + 1 digit	±0.050% + 1 digit
Number of digits	5	5

Two smartphones were used to record the audio: (a) smartphone 1 (T1 in this paper), a model released in 2014 priced at €180 and (b) smartphone 2 (T2 in this paper), a model released in 2017 priced at €249. Two free apps are installed in both mobile phones: (a) Voice recorder, from Splend Apps (App1) [24] and (b) AudioRec, from AC SmartStudio (App2) [25]. These applications allow for the selection of the sampling frequency (8–44 kHz) and for recording in PCM audio format with high quality.
